# The effect of the March 2020 COVID‐19 lockdown on national psychiatric contacts in Denmark: An interrupted time series analysis

**DOI:** 10.1002/brb3.2264

**Published:** 2021-07-02

**Authors:** Jens Peter Hansen, Tessa Quirina Bang van Sas, Marianne Fløjstrup, Mikkel Brabrand, Allan Hvolby

**Affiliations:** ^1^ Mental Health Department Esbjerg University Clinic Mental Health Services in the Region of Southern Denmark Esbjerg Denmark; ^2^ Centre for Mental Health Nursing and Health Research (CPS) Mental Health Services in the Region of Southern Denmark Esbjerg Denmark; ^3^ Unit for Health Promotion Research Department of Public Health University of Southern Denmark Esbjerg Denmark; ^4^ The Research Unit for Emergency Medicine Hospital of South West Jutland Esbjerg Denmark; ^5^ Department of Regional Health Research Centre South West Jutland University of Southern Denmark Esbjerg Denmark; ^6^ Child and Adolescent Psychiatry in the Region of Southern Denmark Esbjerg Denmark

**Keywords:** COVID‐19, interrupted time series analysis, population register, professional‐patient relations, psychiatry

## Abstract

The COVID‐19 pandemic resulted in national lockdowns in several countries. Previous global epidemics led to an increase in the number of psychiatric patients presenting symptoms of anxiety or depression. Knowledge about the impact of early lockdown initiatives during the COVID‐19 pandemic on the number of healthcare interactions is sparse. Contacts in this study include all recorded face‐to‐face (FTF) and virtual treatment interactions between patients and healthcare systems.

**Aim**: To investigate both the impact of the Danish lockdown event on psychiatric patients’ contact with the healthcare system, stratified by type of contact (FTF or virtual) and ICD‐10 diagnosis, and how acute contacts were impacted in the five regions in Denmark.

**Methods**: An interrupted time series analysis was applied to determine the effect of the COVID‐19 lockdown event on the number of contacts with psychiatric hospitals in Denmark, from February 25, 2019 to May 3, 2020. The analyses took a Box‐Jenkins approach to fit an autoregressive integrated moving average (ARIMA) model.

**Results**: Virtual contacts replaced most FTF contacts during the lockdown. For most patient groups, the total number of contacts did not decrease significantly. However, for adult patients diagnosed with ICD‐10 F 0–10, 10–19, and 60–69 and child and adolescent patients diagnosed with F 10–19, 70–79, and 80–89, the number of contacts decreased during lockdown. The number of acute contacts with the psychiatric system decreased significantly in all regions in Denmark during lockdown.

**Discussion**: The Danish healthcare system was forced to introduce innovative tele‐psychiatry to mental health care during the lockdown. Disruption to service delivery was minimized because the resources were in place to sustain the transition from FTF to virtual contacts.

## Graphical abstract

This study examined how the lockdown affected patient with psychiatric illnesses contacts to the health care system and acute contacts to the psychiatry in Denmark. The results show a rapid change in which type of contact patients with psychiatric illnesses have to the health care system with transition from FTF to virtual contacts.

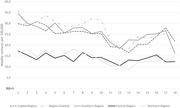

## 1 BACKGROUND

On January 30, 2020, the World Health Organization (WHO) declared the outbreak of COVID‐19 a pandemic (Sohrabi et al., 2020). By the beginning of March 2020, the virus had spread to 72 countries, including Denmark (Sohrabi et al., 2020). Starting from March 11–13, 2020, Denmark locked down most of its national institutions, including health systems, with the exception of critical hospital systems and virtual contacts (Danish Health Authority, 2021). The Danish Health Authority considered mental health services to be critical services. Initiatives to restructure the national health system were implemented, to treat a potentially high number of COVID‐19‐infected patients. The Danish Health Authorities instructed the healthcare directors to extend the existing use of telephone consultations, video consultations, as far as possible (Danish Health Authority, 2020a). However, the low number of face‐to‐face (FTF) contacts after lockdown worried the GPs, who suggested that virtual consultations would be ineffective (Petersen, 2020).

The number of video consultations across the entire Danish healthcare system increased during the period of lockdown; however, the increased activity in virtual contacts was not similar to the reduction that was seen in the number outpatient FTF contacts (Danish Health Authority, 2020b). In mental health services, the number of admitted inpatients and the number of outpatient FTF contacts decreased during the lockdown event, which was also seen in the United Kingdom (Chen et al., 2020a, 2020b). After the end of the March 2020 lockdown on April 14, the average number of admissions to mental health services was the same as before the lockdown (Danish Health Authority, 2020b). The number of virtual contacts increased in the same period (Danish Health Authority, 2020b); however, the reported reduction in total contacts is not specified for psychiatric contacts (Danish Health Authority, 2020b).

Epidemics have previously led to an increase in patients presenting anxiety and depressive symptoms (Kang et al., 2020), and COVID‐19 has led to the same problems in the initial phase of the pandemic (Hyland et al., 2020). During the current COVID‐19 pandemic, Denmark applied a strategy that included an early partial lockdown of the healthcare system, resulting in fewer FTF contacts and reduced aid from municipal services. The effect of those strategies on patients’ need for healthcare is unknown. Thus, it is unclear whether the previously found effects of epidemics (Kang et al., 2020) can be found in the March 2020 lockdown situation, and it is unclear how the presence of COVID‐19 and the partial lockdown of public systems affected the extent of psychiatric problems among citizens in Denmark.

The identified national reduction in number of contacts was for all patient groups. Thus, more research was needed about the impact of early lockdown initiatives on the number of healthcare contacts in mental health services. There was a need for a time series analysis study of changes in number of mental health contacts during the lockdown of mental healthcare system (Chen et al., 2020a, 2020b). This analysis should uncover the impact of the lockdown on psychiatric patients’ acute contacts stratified by psychiatric diagnosis (ICD‐10‐CM blocks).

## 2 AIM

To investigate both the impact of the Danish lockdown event on psychiatric patients’ contact with the healthcare system, stratified by type of contact (FTF or virtual) and ICD‐10 diagnosis, and how acute contacts were impacted in the five regions in Denmark.

## 3 METHODS

An interrupted time series (ITS) analysis (Bernal et al., 2017) was used to investigate the aim.

### 3.1 Study setting

The study includes national Danish data from February 25, 2019 to May 3, 2020 (week 9, 2019 to week 18, 2020). Data regarding all professional–patient contacts between psychiatric patients and psychiatric hospitals, outpatient clinics and general hospitals were included in this study. All data were sourced from the Danish Civil Registration System (DCRS) (Pedersen, 2011). Information on hospital attendance (admission and discharge dates), diagnoses, and procedures were extracted from the Danish National Patient Registry (DNPR; Schmidt et al., 2015). Some of the contacts were reported as acute contacts. An acute contact is defined as a patient contact based on a health condition that requires immediate health intervention (The Danish Health Data Authority, 2019). Most of the acute contacts were to psychiatric emergency departments.

The annual number of patients treated in 2018 by the mental health care system in Denmark was 177,000 (The Danish Health Data Authority, 2020). The five regions are not of equal size. The largest region is the Capital Region, in which 54,251 mental health patients are treated annually, while the smallest is the Region of Northern Jutland, with 16,187 (The Danish Health Data Authority, 2020).

Regional councils are responsible for psychiatric services (Danish Regions, 2012), and psychiatric treatment regimens are based on recommendations from the Danish Health Authority. Mental health services in the five regions are not delivered uniformly. In the Central and Northern Jutland Regions, patients are admitted to acute psychiatric care after referral from their GP or other healthcare departments. In the other regions, patients can receive acute treatment after self‐referral to psychiatric emergency departments. In four out of the five regions, citizens under the age of 18 are treated by child and adolescent psychiatry. In the present study, all contacts from patients under the age of 18 are considered as child and adolescent contacts.

### 3.2 Externally imposed events

The March 2020 lockdown is considered an externally imposed event in this study. Because of the increasing number of COVID‐19 cases and COVID‐19‐related hospitalizations, the Danish Government decided to lock down all usual activities in the Danish healthcare systems from March 13, 2020 (Danish Health Authority, 2020b; The Prime Minister's Office, 2020). The national lockdown of most private and public health‐related services resulted in fewer COVID‐19 cases than expected. Therefore, the government partly re‐opened society and normalized the health system on April 14 (The Danish Health Data Authority, 2020; The Region of Southern Denmark, 2020).

### 3.3 Data collection

Both DCRS and DNPR databases are considered high‐ranking in terms of data quality (Schmidt et al., 2015). DNPR was delivered in a temporary form, made available for COVID‐19 pandemic research by the government, with data from February 25, 2019 until May 3, 2020.

DNPR includes information on individual hospital attendances, and any transfer between hospital units was coded in the database as individual attendances. For the purpose of an assessment of attendance as an entire admission, rather than individual attendances, we combined instances of multiple attendances within four hours into one attendance, which was designated as one contact in the results. Data on the number of inhabitants in Denmark and in the five regions was sourced from Statistics Denmark, which collects, compiles, and publishes statistics on the Danish society (Statistics Denmark, 2021).

### 3.4 Data availability

According to Danish law, data that include sensitive personal information cannot be shared. However, the data sources are available for other researchers, pending approval from the Danish Health Data Authority.

### 3.5 Data analysis

The extracted data consist of two datasets on aggregated weekly contacts. The first dataset consists of contacts to the healthcare system by patients with psychiatric illnesses. The datasets were stratified on ICD diagnoses ICD 10‐CM blocks (Statistics Denmark, 2021), child and adolescent/adult care and for virtual/FT contacts. In the first dataset, we found outliers during public holidays (Easter and Christmas). Thus, we examined holiday effect in all analyses of this dataset. Data on virtual contact was log‐transformed before the analyses, to address the distribution of contacts across weeks. The second dataset consists of acute contacts to the psychiatric system stratified on regions in Denmark (The Danish Health Data Authority, 2020). The data on acute contacts had no seasonality.

The average weekly number of contacts (continuous variables) was compared before and after March 13, 2020, taking the time series structure of the data into account. An ARIMA model analysis is possible and recommended when analyzing aggregated time series data (Bernal et al., 2017; Yao et al., 2020). The Box‐Jenkins approach was applied to fit an autoregressive integrated moving average (ARIMA) model (Lima et al., 2020). The data on virtual contacts were analyzed using an ARIMA model, including autocorrelation AR(1) with robust estimation of the variances. The data on diagnoses were analyzed using an ARIMA model, including autocorrelation AR(1) with robust estimation of the variances. The data on acute contacts were analyzed using an ARIMA model, including moving average MA(1) with robust estimation of the variances.

The ARIMA model was applied because of autocorrelation in the data. The model fit and sensitivity analyses included Bartlett's test, Portmanteau test, Augmented Dickey‐Fuller test, together with an assessment of residuals of the autoregressive models. One region had an increased number of acute virtual contacts after the start of the lockdown. Since none of the other regions included valid data on acute virtual contacts, the analysis on acute psychiatric contacts includes only the data from acute FTF contacts.

### 3.6 Ethics

According to Danish law, observational studies that do not include sensitive personal information do not require ethical approval. The study was approved by the Danish Patient Safety Authority (file no. 20/18426).

## 4 RESULTS

The total number of contacts included in the analyses of diagnoses was 1,796,831 (contacts from patients without diagnosis were omitted). The dataset has 62 weeks of data with 933–1597 weekly acute contacts and 6140–32,759 weekly contacts for all attendances.

### 4.1 Type of contacts for all attendances

**FIGURE 1:**
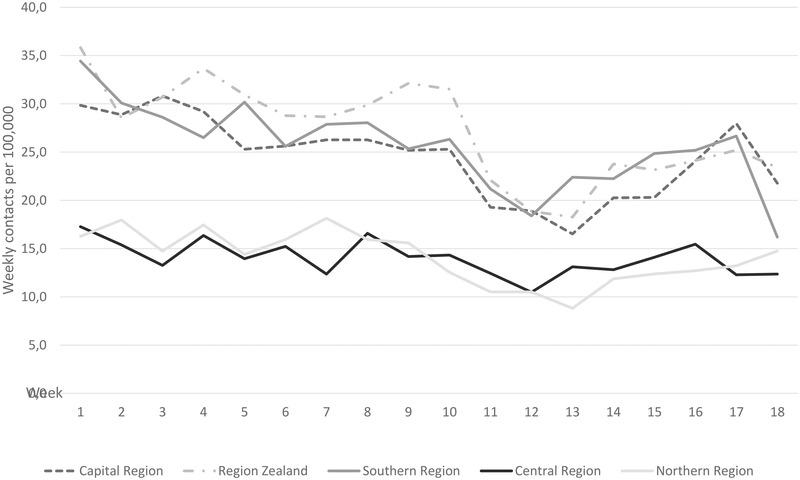
Number of weekly face‐to‐face contacts per 100,000 population in 2020

The type of professional–patient contacts radically changed during the lockdown event. Within one week of the start of the lockdown, the effect was instantaneously altered from FTF to virtual contacts, as illustrated in Figure 1. The number of virtual contacts increased significantly, by 3.30 times, during lockdown (*p* = .0004). The contacts decreased in both FTF and virtual contacts in week 15, in connection with the Easter public holiday. As illustrated in the figure, the number of FTF contacts increased again after the Easter public holiday, to a higher number of FTF than virtual contacts by the end of the observation period.

### 4.2 Adult contacts to the healthcare system stratified by ICD‐10 diagnosis

The total number of contacts (FTF and virtual) from patients did not decrease significantly during lockdown (coefficient = −1092.17, *p* = .4.97), as illustrated in Table 1. However, a sub‐analysis using stratification for diagnosis revealed a lockdown effect for some patient groups. Patients with ICD‐10 diagnosis on mental and behavioral disorders due to psychoactive substance use (ICD‐10 = F10–F19) had a decreased number of weekly contacts (coefficient = −92.99, *p* < .001). Additionally, adult patients suffering from disorders of adult personality and behavior (F60–F69) had a significant decrease in contacts during lockdown. This patient group had a reduced total number of contacts during lockdown (coefficient = −528.51, *p* = .017). We found no significant reduction in total weekly contacts for all other diagnosis groups.

**TABLE 1:** Adult weekly total contacts by diagnosis

Diagnoses	Mean weekly contacts before lockdown	Coefficient	Standard error	*p*‐Value	Confidence interval
F00–F09	1071	−170.0	101.4	.094	[−356.8, −236.8]
F10–F19	638.5	−92.99	26.21	<.001*	[−144.4, −41.6]
F20–F29	7106	576.87	517.93	.265	[−438.3, 1592.0]
F30–F39	4898	−90.47	401.84	.822	[−878.1, 697.1]
F40–F48	4196	−499.27	370.45	.178	[−1225.3, 226.8]
F50–F59	863.2	−162.36	185.04	.380	[−162.4, 200.3]
F60–F69	3018	−528.51	220.76	.017*	[−961.2, −96.8]
F70–F79	215.3	0.79	17.85	.965	[−34.2, 35.8]
F80–F89	250.6	−9.43	17.20	.583	[−43.1, 24.3]
F90–F98	812.5	−38.49	94.19	.683	[−223.1, 146.1]
Total adult contacts	22,369	−1092	1610	.497	[−4247, 2063]

* *p* < .05; ^**^ *p* < .01; ^***^ *p* < .001.

ICD‐10 diagnosis codes:

F00–F09 = Organic, including symptomatic, mental disorders

F10–F19 = Mental and behavioral disorders due to psychoactive substance use

F20–F29 = Schizophrenia, schizotypal and delusional disorders

F30–F39 = Mood [affective] disorders

F40–F48 = Neurotic, stress‐related and somatoform disorders

F50–F59 = Behavioral syndromes associated with physiological disturbances and physical factors

F60–F69 = Disorders of adult personality and behavior

F70–F79 = Intellectual disability

F80–F89 = Pervasive and specific developmental disorders

F90–F98 = Behavioral and emotional disorders with onset usually occurring in childhood and adolescence

The analyzed data includes all contacts (virtual, FTF and other contacts).

### 4.3 Child and adolescent contacts to the healthcare system, stratified by ICD‐10 diagnosis

The total number of contacts with child and adolescent patients for primary care outpatient clinics did not decrease significantly during lockdown (coefficient = −672.51, *p* = .106), as illustrated in Table 2. However, a sub‐analysis using stratification for diagnosis revealed a lockdown effect for some patient groups. In the child and adolescent patient group, we found a different distribution regarding diagnosis, compared to adult psychiatric patients. As for adult patients, we found a decrease in number of contacts in patients treated for substance misuse (ICD‐10 F10–F19). The reduction in weekly contacts was significant (coefficient = −11.56, *p* < .001). Contact with patients with intellectual disability (ICD‐10 F70–F79) and patients with pervasive and specific developmental disorders (ICD‐10 F80–F89) decreased significantly. The contact decreased with a coefficient = −92.99 and a *p*‐value <.001 for ICD‐10 F70–F79 and coefficient = 201.38, *p* = .005 for the ICD‐10 F80–F89 group.

**TABLE 2:** Child and adolescent weekly total contacts by diagnoses

Diagnoses	Mean weekly contacts before lockdown	Coefficient	Standard error	*p* value	Confidence interval
F00–F09	No data				
F10–F19	36.0	−11.56	2.09	.001***	[−15.7, −7.46]
F20–F29	448.6	−19.44	42.51	.644	[−103.0, 63.64]
F30–F39	375.6	−11.57	27.04	.669	[−64.55, 41.42]
F40–F48^a^	851.3	126.36		.07	[263.0, 10.3]
F50–F59	504.1	−10.50	36.52	.774	[−82.1, 61.07]
F60–F69	194.3	−18.06	14.65	.218	[−46.8, 10.66]
F70–F79	52.0	−10.83	5.17	.036*	[−21.0, −0.689]
F80–F89	824.8	201.38	71.87	.005**	[−201.4, −60.53]
F90–F98	1885	200.42	154.08	.193	[−502.4, 101.6]
Total contacts	5668	−672.51	415.70	.106	[−1487, 142.2]

^*^p < .05; ^**^ *p* < .01; ^***^ *p* < .001.

^a^ The numbers are ambiguous due to impact from summer holiday 2019.

ICD‐10 diagnosis codes:

F00–F09 = Organic, including symptomatic, mental disorders

F10–F19 = Mental and behavioral disorders due to psychoactive substance use

F20–F29 = Schizophrenia, schizotypal and delusional disorders

F30–F39 = Mood [affective] disorders

F40–F48 = Neurotic, stress‐related and somatoform disorders

F50–F59 = Behavioral syndromes associated with physiological disturbances and physical factors

F60–F69 = Disorders of adult personality and behavior

F70–F79 = Intellectual disability

F80–F89 = Pervasive and specific developmental disorders

F90–F98 = Behavioral and emotional disorders with onset usually occurring in childhood and adolescence

The analyzed data includes all contacts (virtual, FTF and other contacts).

We found no significant reduction in total weekly contacts for all other diagnosis groups.

### 4.4 Acute contacts

The results from 107,670 contacts showed 1310 mean (SD = 76.4) total number of FTF contacts before lockdown and 1089 mean (SD = 126.6) during lockdown. The mean age was 38.6 (SD = 3.13) with 58.2% women. The number of weekly contacts was different across the regions, with the highest mean contacts per 100,000 inhabitants in the Region of Zealand at 28.3 (SD = 2.89) before lockdown, and lowest in the Central Region, at 14.1 (SD = 1.17), as illustrated in Table 3.

**TABLE 3:** Acute face‐to‐face contacts

Regions	Mean weekly contacts before lockdown	Coefficient	Standard error	*p* value	Confidence interval
Capital Region	487.4	−100.03	25.01	<.001***	[−97.0, −40.0]
Region Zealand	237.3	−50.27	8.02	<.001***	[−30.1, −1.67]
Southern Denmark	339.6	−68.50	14.53	<.001***	[−27.7, −11.3]
Central Denmark	186.6	−15.88	7.25	.029*	[−149.0, −51.0]
Northern Denmark					
	89.7	−19.51	4.18	<.001***	[−65.0, −34.5]
All Denmark	1340	−254.92	45.47	<.001***	[−344.0, −165.8]

^*^p < .05; **p < .01; ^***^ *p* < .001.

**FIGURE 2:**
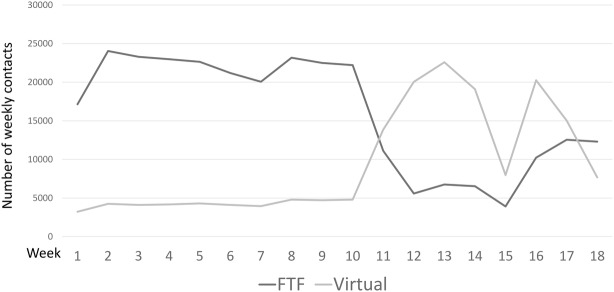
Number of national weekly face‐to‐face and virtual contacts in 2020

The number of weekly acute contacts decreased significantly in all regions of Denmark. The national weekly contacts decreased from 23.0 (SD = 1.31) contacts per 100,000 inhabitants to 18.7 (SD = 2.17) *p* < .001. The number of weekly contacts decreased after March 13, as illustrated in Figure 2. The Central Region had the lowest difference in coefficient, at −15.88, indicating a minor effect of lockdown in this region.

## 5 DISCUSSION

The results showed that, in most cases, FTF contact was replaced by virtual contact during lockdown in Denmark. The total number of contacts to the health system in relation to patients with psychiatric illnesses did not decrease significantly during the lockdown. However, not all diagnosis groups received the same number of contacts during transition to virtual contacts. The result showed a significant decrease in the number of acute weekly contacts.

The COVID‐19 pandemic has disrupted and otherwise impacted mental health services in most countries, including higher levels of psychological distress among the general population (Lima et al., 2020) and greater symptom exacerbation among patients with mental health problems (Yao et al., 2020). In many countries including Denmark, the pandemic has resulted in the application of digital solutions to replace FTF contact (Golinelli et al., 2020). The number of virtual contacts increased immediately after the Danish government directed the health systems to transfer from FTF contact to virtual contact. The change in type of contact in mental health is similar to the transition to virtual contacts during lockdown in the entire Danish health system (Danish Health Authority, 2020c). In the report from the Danish Health Authority on activities in Denmark during lockdown, a significant increase in virtual contacts is reported from week 11, and the number decreased again from week 17 (Danish Health Authority, 2020c). We found an outlier in week 18, showing a low number of virtual contacts, which is similar to the low number of virtual contacts at this time in the entire health system, as reported in the national report on activities (Danish Health Authority, 2020b). The number of virtual contacts decreased further in the following weeks, across the entire health system (Danish Health Authority, 2020b). Thus, the low number we found in week 18 probably reflects the normalization of the mental health system. The major change in form of contact is in line with the recommendations by the United Nations on scaling up access to remote support when people are forced to maintain social distance during the COVID‐19 pandemic (United Nations, 2020). The pandemic forced the Danish healthcare system to extend the existing innovative tele‐psychiatry to mental health care (United Nations, 2020). The Danish society had the infrastructure and resources to transition to virtual contact in periods of high‐risk exposure in connection with pandemics (World Health Organization, 2020b).

The lockdown did not have a significant impact on contact between patients with psychiatric illnesses and healthcare services. However, the results indicate that patients are affected, differently depending on their ICD‐10 diagnosis. Most of the diagnosis groups received the same number of contacts during lockdown compared to before lockdown. The finding follows the trend from other regions around the world and the recommendation to increase the use of tele‐psychiatry and other digital health interventions (Kozloff et al., 2020).

We found that two groups of patients were significantly affected by the lockdown, as illustrated in Table 2. Patients suffering from substance abuse had a decrease in total number of contacts to the healthcare system. The decrease in treatment contacts might come from reduced treatment needs during a lockdown, with reduced contacts and access to centers for treatment of drug and alcohol addiction/dependency. It is crucial to determine whether those patients are unstable and need help, or whether they are in good shape with a better situation and with fewer met or unmet needs. (Lin et al., 2020)

There was a reduction in the number of contacts during lockdown with adult patients suffering from personality and behavioral disorders. Research on the effect of COVID‐19 regarding this group is sparse. In Denmark, the preferred treatment for this group of patients is group treatment. The decrease we found in the number of contacts could be explained by the fact that a number of patients did not receive an alternative individual treatment to their usual FTF contact. During the lockdown, group sessions were cancelled. Another possibility is that challenges were experienced in transitioning from FTF to virtual treatment for this specific patient group.

We found no significant reduction in the number of contacts for patients suffering from dementia and similar diseases. However, we found a trend toward fewer contacts during lockdown. People suffering from dementia are vulnerable in more areas. They are at higher risk for serious COVID‐19 symptoms because of their generally older age and greater number of comorbidities (Ryoo et al., 2020). Additionally, these patients are at a risk of deterioration of their dementia (Ryoo et al., 2020). Thus, it is important during a lockdown to supervise the assistance provided to patients suffering from dementia.

The results did not show a significant effect of lockdown on contact with patients suffering from anxiety. This is in contrast to previous studies (Kang et al., 2020) from Wuhan, which showed an increase in the number of patients presenting with symptoms of anxiety. However, we only investigated the first weeks of lockdown. It could be that referrals of patients with severe anxiety or depressive symptoms might have been delayed during the period under investigation in the present study. Additionally, the number of people infected by COVID‐19 was relatively low (cumulative incidence = 188.6, May 19, 2020) (Statens Serum Institut, 2020) at the time of study.

Vulnerable children with diagnosis ICD‐10 F80–F89, such as children with autism spectrum disorder (ASD), would be expected to be of particular concern. Their main difficulties are characterized as challenges in social communication, social interaction, and reciprocal behavior (World Health Organization, 2020a). They often need a predictable environment and may react to changes in daily routines with more stress, anxiety or worsening of core symptoms (Baron‐Cohen, 2006). In this regard, a study as expected found an increase in difficulties among children and adolescents with ASD (Colizzi et al., 2020). We found, on the contrary, that this group had fewer contacts. This could partly be explained by the fact that, in the first weeks after lockdown, there was some confusion among health care providers about the best way to deal with the new situation. ASD patients primarily have contact with outpatient departments regarding tests and diagnoses, so in most cases it is necessary to see the patient FTF to carry out the tests that are included in the diagnosis process. Furthermore, treatment often takes the form of psychoeducational activities performed in groups, which were cancelled during the lockdown.

For child and adolescent patients with intellectual disability diagnosis (ICD10 F70–F79), the reason for the decrease in the number of contacts with the healthcare system is less certain. The explanation might be that, in spite of the generally negative reaction to lockdown and isolation (Lima et al., 2020; Yao et al., 2020), this group did benefit during the first weeks of lockdown, when the cancellation of school activities might have resulted in less stress.

For the group with substance abuse, as for adults, the reduced number of treatment contacts might come from a decrease in need for treatment during the lockdown, related to reduced contacts and access to centers for treatment of drug and alcohol addiction/dependency.

The decrease in acute contacts is in accordance with the decrease in the number of admissions to psychiatric departments in all Danish regions in the same period, as illustrated in a national report (Danish Health Authority, 2020b). The number of admissions increased again after the reopening of the society on April 14 (Danish Health Authority, 2020b), and we can see a similar trend in Figure 3, showing a small increase in the number of acute contacts. The decrease in acute contacts shows a similar pattern to the sharp reduction in “front door” mental health and acute referrals found in Cambridgeshire and Peterborough, during the UK lockdown (Chen et al., 2020a, 2020b). In our present study, the number of acute contacts decreased most in the region with the highest number of weekly contacts per 100,000. Thus, there can be a trend that some people discontinued contacting psychiatric hospitals during the Danish lockdown.

### 5.1 Limitations

This study has several limitations. The results were sourced from aggregated data, which impedes explanatory results. Furthermore, the data are aggregated weekly without accounting for individual patients’ influence. Time series analyses of aggregated data should include more than 50 time points (Zhang et al., 2011). The data in this study includes 62 time points (weeks). However, the number of time points should ideally be the same before and during the event. The set of data includes 54 time points before and eight time points during the lockdown event. Thus, the results with low effect size should be considered with caution, and the results that are significant might have appeared as trends in this study. Furthermore, we found an effect from public holidays on all contacts. One of the public holidays was Easter, 2020. We adjusted for the public holidays in the analysis. Since one of the time points is during lockdown, it results in an additional limitation regarding the results. The effect of lockdown on acute contacts with psychiatry services might have initiated more use of virtual contacts. However, the number of acute virtual contacts is diversely documented in the Danish regions. Thus, we omitted the analysis of those data. Furthermore, we omitted patients without a diagnosis in the analysis of total number of contacts.

A partial lockdown of the Danish healthcare system led to several challenges (Abdullah et al., 2021). In a very short space of time, both patients and care providers had to change their usual referral methods and forms of contact. The results show a rapid change in the type of contact patients with psychiatric illnesses in the transition from FTF to virtual contacts. We suggest further research on regional differences and between patient groups and on the increase in contacts after March 3 for various patient groups, and to investigate the development of contacts after re‐opening the society and the healthcare system. These results from the present study highlight a need to investigate the effect on various patient groups over a longer period. Additionally, such studies might reveal the effect on other patient groups during a long lockdown rather than in the acute phase. The long period of reduced social interaction from March 2020 to April 2021 might have an impact, such that more people present with symptoms of anxiety and depression. There is a need for knowledge on the patient characteristics of those psychiatric patients who are affected by either more or fewer contacts during lockdown. The changes in the type of contacts to the healthcare system might decrease the number of contacts for some patient groups and other patient groups might have an increased need for care in connection with an increase in psychiatric symptoms during lockdown.

### PEER REVIEW

The peer review history for this article is available at https://publons.com/publon/10.1002/brb3.2264.

## Data Availability

The data that support the findings of this study are available on request from the corresponding author. The data are not publicly available because of privacy‐based or ethical restrictions.
